# Current status and influential factors associated with adherence to self-monitoring of blood glucose with type 2 diabetes mellitus patients in grassroots communities: a cross-sectional survey based on information-motivation-behavior skills model in China

**DOI:** 10.3389/fendo.2023.1111565

**Published:** 2023-06-27

**Authors:** Manxin Lin, Tingting Chen, Guanhua Fan

**Affiliations:** ^1^ School of Public Health, Shantou University, Shantou, China; ^2^ Shantou University Medical College, Shantou, China; ^3^ Health Management Center of Outpatient Department, Cancer hospital of Shantou University Medical College, Shantou, China

**Keywords:** type 2 diabetes mellitus (T2DM), pre-diabetes, self-monitoring of blood glucose (SMBG), adherence, influencing factors, IMB model

## Abstract

**Objective:**

Self-monitoring of blood glucose (SMBG) plays a vital role in the maintenance of blood glucose with type 2 diabetes mellitus(T2DM) and pre-diabetes patients. The study was intended to describe the current status of SMBG with T2DM and pre-diabetes patients in grassroots communities, explore the relationship between SMBG frequency and blood glucose level and apply information-motivation-behavior(IMB) model to analyze the potential influencing factors of SMBG compliance based on electronic questionnaires.

**Methods:**

A cross-sectional study was conducted with 1388 T2DM and pre-diabetes patients who completed electronic questionnaires composed of demographics and IMB model content. Chi-square test, Mann-Whitney U test and multivariable logistic regression model analysis were utilized to explore deeply causes of SMBG compliance.

**Results:**

The results of this study showed that among 1388 T2DM patients, only 26.2% (363/1388) patients reached SMBG standard, indicating low compliance with SMBG. Given that SMBG is one of the individual predictors of type 2 risk in prediabetes patients, this result suggests that the SMBG compliance rate needs to be improved. Patients with fixed occupation (OR=1.989, *P*=0.035), BMI in normal range (OR=1.336, *P*=0.049), smoking habit(OR=1.492, *P*=0.019), understanding SMBG frequency (OR=1.825, *P*<0.001), understanding control goal of blood glucose (OR=1.414, *P*<0.001), knowing all the functions of the blood glucose meter (OR=1.923, *P*<0.001), buying a blood glucose meter/test paper conveniently(OR=2.329, *P*=0.047), taking supplementary measurement when forgetting blood glucose test(OR=2.044, *P*=0.005), rotating all the fingers when measuring blood glucose (OR=1.616, *P*<0.001) and less pain at the needling site(OR=2.114, *P*<0.001)were independently promoting factors of adherence to SMBG. However, the lack of accessibility and convenience of blood glucose meter or heavy financial burden were blocking factors of adherence to SMBG. Moreover, there were still bottlenecks such as lack of health care knowledge and needle pricking pain.

**Conclusion:**

This study verified the practicability of applying IMB model to SMBG with T2DM and pre-diabetes patients. Adherence to SMBG still remained to improved, and putting more emphasis in improvement of individual information, motivation and behavioral skills with patients might be beneficial to maintain better adherence to SMBG in long-term routine of diabetes self-management.

## 1 Introduction

In recent years, the incidence and prevalence rate of DM has increasing worldwide, with its protracted courses and high mortality rate, which has placed a heavy burden on health of the global population. Under the background of primary care, community is the “front line” and “main battlefield” of diabetes management. Diabetes management in grass-roots communities is one of the most effective means to delay the progress of diabetes and reduce the risk of diabetes complications (1). At present, there are many deficiencies in diabetes management in domestic diabetes communities, such as inconsistent standards, low operating efficiency, floating form and so on (2, 3). The keys to integrate respective advantages, improve patients’ cognition of diabetes, reduce patients’ economic burden as much as possible and maximize the convenience and accessibility of community health resources at the grass-roots level is to exert the synergy of self-monitoring in the comprehensive management of diabetes. Tilting the diabetes management mode to the community, taking the self-monitoring of diabetics in the daily community environment as a breakthrough, and digging out a replicable self-monitoring mode for diabetics in the community will give new significance to the community management of diabetes in the post-epidemic era. Admittedly, grass-roots communities shoulder the heavy responsibility of diabetes management.

According to the latest data from the International Diabetes Federation, an estimated 537 million adults aged 20-79 were living with diabetes in 2021, accounting for 10.5 percent of the global population. China has about 140 million diabetics (about 26% of the world’s total). T2DM was the most common type of diabetes(90% of all diabetics), whose prevalence rate has increasing year by year and the epidemic trend has not yet reached the plateau. In addition, pre-diabetes was regarded as a key window period, which was a sign or watershed to reverse diabetes (4, 5). Around 5%-10% of pre-diabetes turned into diabetes each year. In 2003, American Diabetes Association (ADA) proposed that a critical threshold for diagnosing of pre-diabetes impaired fasting blood glucose (IFG) should be reduced from 6.1 mmol/L to 5.6 mmol/L. It is estimated that the scale of people with IFG in China will increase to exceed to 470 million and more and more people have become a huge reserve army for diabetes (6). Move the strategic pass of diabetes management forward could make more patients be managed at an early stage, in order to reduce diabetes complications.

Diabetes is a typical lifestyle disease, and its prevention and control needs comprehensive strategy of “Five Carriages of Diabetes Treatment”(FCDT), which is composed of drug treatment, blood glucose monitoring, diet control, exercise control and health education. Single continuously intensive drug treatment was no longer the priority strategy (7). Among them, blood glucose monitoring played a vital role in controlling blood glucose and inhibiting complications. As an effective tool for diabetes management, SMBG could help patients better understand their own conditions and then making corresponding drug and behavior adjustments. On the other hand, it could also provide more diagnosis and treatment basis for clinicians, so that the disease conditions of diabetics could be better controlled (8–10).

Many studies turned out that there was a yawning gap between recommended SMBG frequency and the actual application, which were affected by many factors (11). Studies in China, the United States, Europe and worldwide have revealed several factors that affect patients’ compliance with SMBG, including occupation, economic status, living habits, hobbies, SMBG related cognition, SMBG related behaviors, accessibility and so on. Additionally, a series of studies exhibited that the practicability and maturity of IMB model have been verified on T2DM. Integrating analysis of various influencing furtherly was beneficial to provide a clearer explanation of ultimate causes and feasible goals of SMBG behaviors (12, 13). Research have shown that the increase of diabetes knowledge (information) was associated to the significantly reduced glycosylated hemoglobin level, which contributed to the self-management of diabetes (14–16). High motivation level improved T2DM patients SMBG compliance (17). And good behavioral skills were essential to enhance SMBG compliance which was key to reach the best blood glucose level (18, 19). Nevertheless, most studies neglected the influence from patients in pre-diabetes stage, as well as failed to fully explore the relationship between IMB model and SMBG of T2DM patients, which was exactly the gap that our study intended to fill with.

In a nutshell, the study selected T2DM and pre-diabetes patients in China grassroots communities as research objects, explored status of SMBG based on the IMB framework and analyzed the influencing factors of adherence to SMBG, deeply explaining the maintenance mechanism and providing a new sight of SMBG with T2DM and pre-diabetes patients.

## 2 Objects and methods

### 2.1 Research design and data collection process

#### 2.1.1 Research design and participants

The subjects of the study were diabetic patients with different clinical stages available to the investigator’s unit (Shantou community). The data collection method of convenience sampling was adopted in this study, and quality control was strictly implemented in the process to ensure good quality of data provided by diabetes patients in grassroots communities participating in this study.

**Figure 1:**
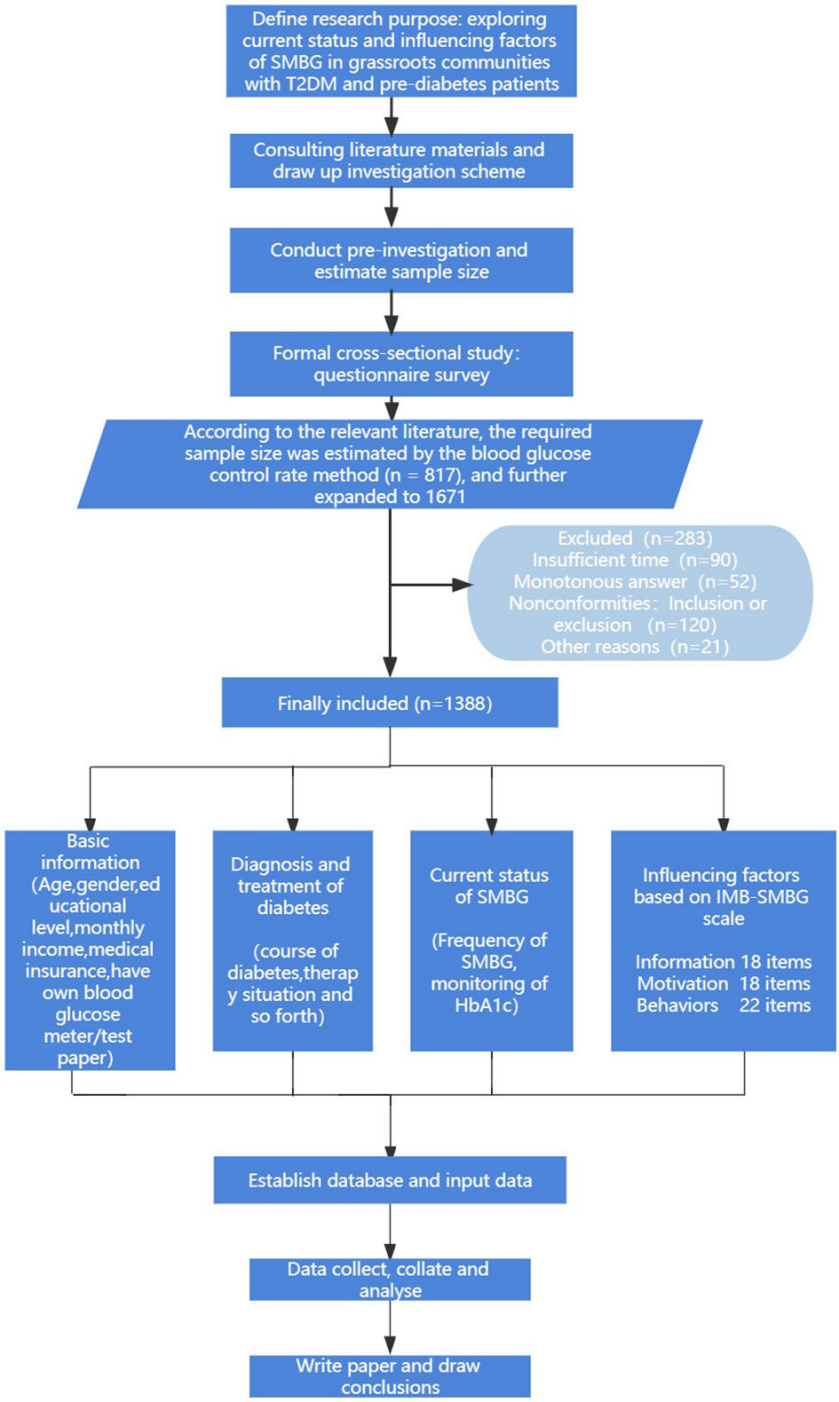
Study design.

During the study, relevant questionnaires were filled in after obtaining the consent of community patients. Inclusion criteria were: ① Patients with prediabetes and T2DM meeting WHO diagnostic criteria. ② The patient is over 18 years old and has certain understanding and communication skills. ③ The patient had a daily community residence and a relatively stable living state. ④ I have been followed up in the outpatient clinic for diabetes evaluation, and basically know my own diabetes clinical stage. ⑤All the interviewed patients voluntarily participated in this study with informed consent. Exclusion criteria: ① suffering from serious organic disease; ② Patients with severe complications. As shown in **Figure 1**.

#### 2.1.2 SMBG criteria for grouping

According to the standards of China’s guidelines for the prevention and treatment of type 2 diabetes mellitus (2020 edition) (part I), the patients treated with insulin were divided into groups (18). Since patients requiring insulin therapy have different requirements for SMBG frequency than non-insulin therapy patients, we used insulin therapy and non-insulin therapy as the basis for grouping. At the same time, patients were further grouped based on self-reported HbA1c values. As a first step, screening should be performed in patients treated with insulin, when HbA1c is in the normal range, the frequency of SMBG should be 4 or more times per day. If the HbA1c value is not within the normal range, the SMBG frequency should be 7 or more times per day. Next, in patients not receiving insulin, the frequency of SMBG should be 6 or more times per week when HbA1c is within the normal range. If the HbA1c value is not within the normal range, the SMBG frequency should be 4 or more times per day. Finally, all patients with SMBG reaching the required monitoring frequency were included in the SMBG standard group. Patients whose monitoring frequency did not meet the requirements were included in the SMBG sub-standard group. As shown in **Table 1**.

**Table 1:** SMBG grouping standard.

	HbA1c normal value	HbA1c abnormal value
Insulin therapy	≥4 times/day	≥7 times/day
Non insulin therapy	≥6 times/week	≥4 times/day

The normal range of glycated hemoglobin in patients with type 2 diabetes is 4.4% ~ 7.0%, and that in patients with pre-diabetes is less than or equal to 6.1% (20).

### 2.2 Methods

#### 2.2.1 Research indicators and tools

The IMB-SMBG scale (21) was used for quantitative statistics, and the IMB-SMBG scale was translated into the Chinese version. The Chinese version of the IMB-SMBG questionnaire consisted of three subscales: SMBG information, motivation and behavioral skills. This questionnaire has a total of 58 items, including 18 items in information part, 18 items in motivation part and 22 items in behavior part. This is a five-level Likert scale with five responses: “strongly agree”, “agree”, “neutral”, “disagree” and “strongly disagree”. The score of each item ranges from 1 to 5. The compliance degree of blood glucose self-monitoring of each patient will be the total score of the sum of the scores obtained from the answers to each question. The total score of this part represents the compliance degree of blood glucose self-monitoring of the patient, and the higher score represents the better diabetes self-management. The Cronbach’s α coefficient of the questionnaire internal consistency test was 0.965, and the Cronbach’s α coefficients of information, motivation and behavior were 0.695, 0.932 and 0.965, respectively. Construct validity KMO=0.967, Bartlett’s spherical test χ2 = 15299.427, P<0.000.

#### 2.2.2 Sample size

Based on a review of previous literature (22–24), we found that the reported glycemic control rate (HbA1c<7%, 58mmol/mol) in Chinese patients with T2DM varied from 31.78% to 39.7%. Assume that the glycemic control rate is 35%, α is 0.05, and the allowable error of sampling is 0.06. We calculate the required sample size of 1002, which would allow for a non-response rate of 20%, and calculate the required sample size of 1253. In order to further improve the robustness of the research conclusions, we further expanded the sample size. We received 1,671 total questionnaires and excluded 283 invalid questionnaires (such as taking too short time to fill in the questionnaire or giving a single answer), and finally included 1388 sample sizes.

#### 2.2.3 Data collection

The Chinese version of IBM-SMBG scale was input into the electronic questionnaire, and patients filled in the questionnaire by scanning the QR code of “Questionnaire Star Platform”. The purpose and significance of the survey and the method of filling out the questionnaire were explained to the patients through subheadings. All the questionnaires were checked and screened by professionals after they were collected. Records of the patient’s general condition (age, gender, educational level, income, type of medical insurance and have glucose meter/blood sugar test paper, etc.), the situation of diagnosis and treatment of diabetes (course, treatment, etc.), SMBG status (including frequency of SMBG, outpatient measuring blood sugar, glycosylated hemoglobin HbA1c) monitoring and potential influence factors of SMBG.

#### 2.2.4 Statistical analysis

The collected data were analyzed and processed by SPSS25.0. The background data and scale scores were statistically described to present demographic and other characteristics. The measurement data of non-normal distribution was expressed by M(P50), and Mann-Whitney U test was used for comparison between the two groups. The data were expressed in relative number (%), and comparison between the two groups was performed by chi-square test. Multivariate Logistic regression was used to analyze the potential influencing factors of SMBG in T2DM patients. The test level α=0.05, P < 0.05 was considered statistically significant.

## 3 Results

### 3.1 Basic information

A total of 1671 questionnaires were distributed and collected. Excluding the invalid questionnaires such as incomplete filling and short filling time, there were 1388 questionnaires were valid, and the recovery rate was 83.1%(1388/1671). Among them, there were 986 males (71.0%) and 402 females (29.0%). 72% were T2DM patients and 28% were pre-diabetes patients. In China, the number of pre-diabetes patients with low fasting blood glucose and high postprandial blood glucose was huge, hence we included pre-diabetes patients in communities. Moreover, patients who were in pre-diabetes stage could inhibit developing into T2DM by changing their dietary habits and increasing physical exercise. Thus, it was of great value for diabetics to strengthen the adherence to SMBG. Specifically, based on the IMB model composed of the information, motivation and behavior three parts, the score range of the SMBG up-to-standard group were 32-67 (M = 53), 29-86 (M = 61) and 31-110 (M = 73) respectively, while the score range of the SMBG not-up-to-standard group were 31-68 (M = 47), 22-86 (M = 50) and 22-88 (M = 59) respectively, with differences statistically significant (P < 0.001). As shown in **Table 2**.

**Table 2:** Bivariate analysis of potential influencing factors of SMBG.

	SMBG adherence (n=363)	SMBG non-adherence (n=1025)	χ^2^/Z value	P value
Age [n (%)]				
Under 18 years old	5 (20.8)	19 (79.2)	1.873	0.866
18-30 years old	144 (26.9)	391 (73.1)		
31-45 years old	127 (24.8)	386 (75.2)		
46-60 years old	73 (28.3)	185 (71.7)		
61-75 years old	12 (25.0)	36 (75.0)		
Over 76 years old	2 (20.0)	8 (80.0)		
Gender [n (%)]				
Male	258 (26.1)	728 (73.9)	0	1
Female	105 (26.2)	297 (73.8)		
Type of diabetes [n (%)]				
Pre-diabetes	115 (29.6)	273 (70.4)	3.389	0.067
T2DM	248 (24.8)	752 (75.2)		
Education level [n (%)]				
Primary school and below	8 (19.0)	34 (81.0)	5.834	0.120
Middle school/technical secondary school	98 (28.7)	243 (71.3)		
Colleges	169 (27.6)	443 (72.4)		
Bachelor degree or above	88 (22.4)	305 (77.6)		
Occupation [n (%)]				
Worker	107 (21.8)	384 (78.2)	19.684	0.001^**^
Farmer	130 (26.7)	357 (73.3)		
Freelancer	107 (34.6)	202 (65.4)		
Retired/unemployed	10 (16.4)	51 (83.6)		
cadre	9 (22.5)	31 (77.5)		
Medical fee payment method [n (%)]				
Urban medical insurance	78 (22.6)	267 (77.4)	10.610	0.600
Employee medical insurance	120 (25.2)	357 (74.8)		
Rural cooperative medical insurance	118 (27.3)	315 (72.7)		
Government/business subsidy	28 (40.6)	41 (59.4)		
Diabetes special clinic	14 (29.8)	33 (70.2)		
Self-payment	5 (29.4)	12 (70.6)		
Marital status [n (%)]				
Single	75 (20.3)	294 (79.7)	10.751	0.005^**^
Married	270 (27.8)	700 (72.2)		
Divorced/widowed	18 (36.7)	31 (63.3)		
Duration of diabetes [n (%)]				
<1 year	42 (18.8)	182 (81.3)	24.133	0.000^***^
1-5 years	169 (23.6)	548 (76.4)		
6-10 years	119 (33.3)	238 (66.7)		
11-20 years	31 (37.8)	51 (62.2)		
>20 years	2 (25.0)	6 (75.0)		
Taking oral drugs to control blood glucose [n (%)]				
Yes	218 (25.9)	625 (74.1)	0.95	0.755
No	145 (26.6)	400 (73.4)		
Average monthly income [n (%)]				
<3000	26 (17.2)	125 (82.8)	14.169	0.007^**^
3000-6000	124 (24.5)	383 (75.5)		
6000-9000	144 (27.4)	382 (72.6)		
9000-12000	57 (35.0)	106 (65.0)		
>12000	12 (29.3)	29 (70.7)		
Smoking [n (%)]				
Yes	228 (28.4)	574 (71.6)	5.096	0.026^*^
No	135 (23.0)	451 (77.0)		
Drinking [n (%)]				
Yes	262 (27.8)	679 (72.2)	4.321	0.043^*^
No	101 (22.6)	346 (77.4)		
BMI [n (%)]				
BMI <18.5	51 (24.2)	160 (75.8)	3.061	0.382
18.5≤ BMI <24.0	226 (27.7)	590 (72.3)		
24.0≤ BMI <28.0	60 (22.7)	204 (77.3)		
BMI ≥28.0	26 (26.8)	71 (73.2)		
With diabetic family history [n (%)]				
Yes	173 (27.8)	450 (72.2)	1.529	0.220
No	190 (24.8)	575 (75.2)		
Diabetes-related complications [n (%)]				
Yes	190 (24.9)	573 (75.1)	1.373	0.244
No	173 (27.7)	452 (72.3)		
Outpatient frequency per year [n (%)]				
0-2	107 (21.7)	386 (78.3)	16.966	0.000^***^
3-5	219 (27.1)	588 (72.9)		
≥6	37 (42.0)	51 (58.0)		
Frequency of hospitalizations per year [n (%)]				
<1	94 (24.7)	286 (75.3)	2.544	0.280
1-2	237 (26.0)	673 (74.0)		
>3	32 (32.7)	66 (67.3)		
Knowing HbA1c [n (%)]				
Yes	198 (23.7)	638 (76.3)	6.633	0.011^*^
No	165 (29.9)	387 (70.1)		
Having own blood glucose meter [n (%)]				
Yes	288 (25.7)	831 (74.3)	0.516	0.487
No	75 (27.9)	194 (72.1)		
Knowing the frequency of SMBG [n (%)]				
Yes	79 (33.5)	157 (66.5)	7.893	0.006^**^
No	284 (24.7)	868 (75.3)		
IMB-SMBG scale (information)	53 (32,67)	47 (31,68)	-14.775	0.000^***^
IMB-SMBG scale (motivation)	61 (29,86)	50 (22,86)	-16.430	0.000^***^
IMB-SMBG scale (behavior)	73 (31,110)	59 (22,88)	-17.037	0.000^***^

SMBG, Self-monitoring of blood glucose; BMI, Body mass index.*P<0.05; **P<.01; ***P<0.001.

### 3.2 Bivariate analysis of potential influencing factors of SMBG

There were 363 cases (26.2%) in SMBG up-to-standard group and 1025 cases (73.8%) in SMBG not-up-to-standard group in study. Patients with different occupational status had distinct SMBG compliance, and the difference was significant(*P*<0.001). The study results exhibited that unmarried patients had lower adherence to SMBG than married, divorced or widowed T2DM patients, and the difference was statistically significant (*P*<0.005). As Pereira MG (25) studied, better family coping and higher level of positive support in patient evaluation predict would insist SMBG more after four months of intervention, thus patients with families meant better blood glucose monitoring. As for the course of diabetes, the SMBG compliance of diabetic patients in distinct course of diabetes was different(*P*<0.001). Besides, compared with patients who lacked basic knowledge of diabetes as well as not formed a scientific method of blood glucose control in the diagnosis period (less than half a year after diagnosis of diabetes) and exploration period (six months to two years after diagnosis of diabetes), most patients in the stable period of diabetes (more than two years after diagnosis) had better compliance of SMBG. The SMBG compliance rate in patients with various average monthly income was different (*P*<0.007); patients with smoking and drinking habits had higher SMBG compliance rate, and difference was statistically significant (*P*<0.05); Adherence to SMBG of patients with distinct outpatient frequency per year was different. Patients who went to diabetes clinic more than 6 times a year had better compliance of SMBG, and difference was statistically significant (*P*<0.001). No statistically significant differences in adherence to SMBG, age, gender, type of diabetes, education level, type of medical insurance, taking oral drugs to control blood glucose, BMI, family history of diabetes, having diabetes-related complications, frequency of hospitalizations per year, having own blood glucose meter were found (P>0.05, as shown in **Table 2**).

### 3.3 Multivariate logistic regression analysis of potential influencing factors of SMBG

Whether SMBG meets the standard (yes =1, no =0) is taken as the dependent variable, and factors with statistical significance in the above univariate analysis results are taken as independent variables. As shown in **Table 3**. The results of multivariate Logistic regression analysis revealed that occupational type, BMI, smoking habit, whether knowing frequency of SMBG, whether you feel pain at the prick site, whether knowing all the functions of blood glucose meter, whether buying test paper conveniently, whether understanding self-glycemic control goals, whether taking supplementary measurement when forgetting blood glucose test and whether rotating all the fingers when measuring blood glucose were potential influencing factors of SMBG.(*P*<0.05, as shown in **Table 4**). The compliance of SMBG with T2DM patients in normal BMI range was higher than that in patients without in normal BMI range (OR=1.336, *P*<0.049). Types of occupation could also affect SMBG compliance (OR=1.989, *P*<0.035), and patients with fixed occupation had higher compliance of SMBG than that with freelancers. In addition, the compliance of T2DM patients who knew frequency of SMBG was higher than that of those who knew it less (OR=1.825, *P*<0.001); T2DM patients who had their glucose control goal had higher adherence to SMBG(OR=1.414, *P*<0.001), and knowing all the functions of blood glucose meter was beneficial to improve compliance of SMBG(OR=1.923, *P*<0.001).Therefore, mastering SMBG skills could enhance the understanding of blood glucose monitoring, meanwhile, improving the operability of SMBG could promote adherence to SMBG.

**Table 3:** Assignment of multivariate logistic regression analysis of potential influencing factors of SMBG with T2DM and pre-diabetes patients.

Variable	Value
Gender	Male=1;Female=0
Age	>45 years old=1;≤45 years old=0
Type of diabetes	T2DM patients=1;Pre-diabetes patients=0
Educational level	Bachelor degree or above=1; Blow bachelor degree=0
Occupation	Fixed occupation=1;Freelancer=0
BMI	In normal range=1; out of normal range=0
Whether smoking	Yes=1;No=0
Whether drinking	Yes=1;No=0
Whether knowing the frequency of SMBG	Yes=1;No=0
Whether feeling pain at the needling site	Yes=1;No=0
Whether knowing all the functions of the blood glucose meter	Yes=1;No=0
Whether buying a blood glucose meter/test paper conveniently	Yes=1;No=0
Whether understanding self-glycemic control goals	Yes=1;No=0
Whether taking supplementary measurement when forgetting blood glucose test	Yes=1;No=0
Whether rotating all the fingers when measuring blood glucose	Yes=1;No=0

**Table 4:** Multivariate logistic regression analysis of potential influencing factors of SMBG with T2DM and pre-diabetes patients.

Factors	β	SD	Wald	P Value	OR (95%CI)
Gender	-0.167	0.172	0.937	0.333	0.847 (0.604～1.186)
Age	0.06	0.176	0.001	0.971	1.006 (0.713～1.420)
Educational level	-0.656	0.469	1.955	0.162	0.519 (0.207～1.301)
Occupation	0.688	0.327	4.421	0.035^*^	1.989 (1.048～3.777)
BMI	0.290	0.147	3.874	0.049^*^	1.336 (1.001～1.784)
Whether smoking	0.400	0.170	5.542	0.019^*^	1.492 (1.069～2.081)
Whether drinking	-0.197	0.180	1.191	0.275	0.821 (0.577～1.170)
Whether knowing the frequency of SMBG	-0.602	0.178	11.371	0.001^**^	1.825 (1.287～2.589)
Whether feeling pain at the needling site	0.748	0.176	18.032	0.000^***^	2.114 (1.496～2.986)
Whether knowing all the functions of the blood glucose meter	0.654	0.172	14.526	0.000^***^	1.923 (1.374～2.691)
Whether buying a blood glucose meter/test paper conveniently	0.845	0.166	25.794	0.047^*^	2.329 (1.681～3.227)
Whether understanding self-glycemic control goals	0.346	0.174	3.954	0.000^***^	1.414 (1.005～1.989)
Whether taking supplementary measurement when forgetting blood glucose test	0.715	0.167	18.324	0.005^**^	2.044 (1.473～2.835)
Whether rotating all the fingers when measuring blood glucose	0.480	0.171	7.913	0.000^***^	1.616 (1.157～2.258)

*P<0.05; **P<0.01; ***P<0.001.

### 3.4 ROC curve analysis based on IMB-SMBG score

**Figure 2:**
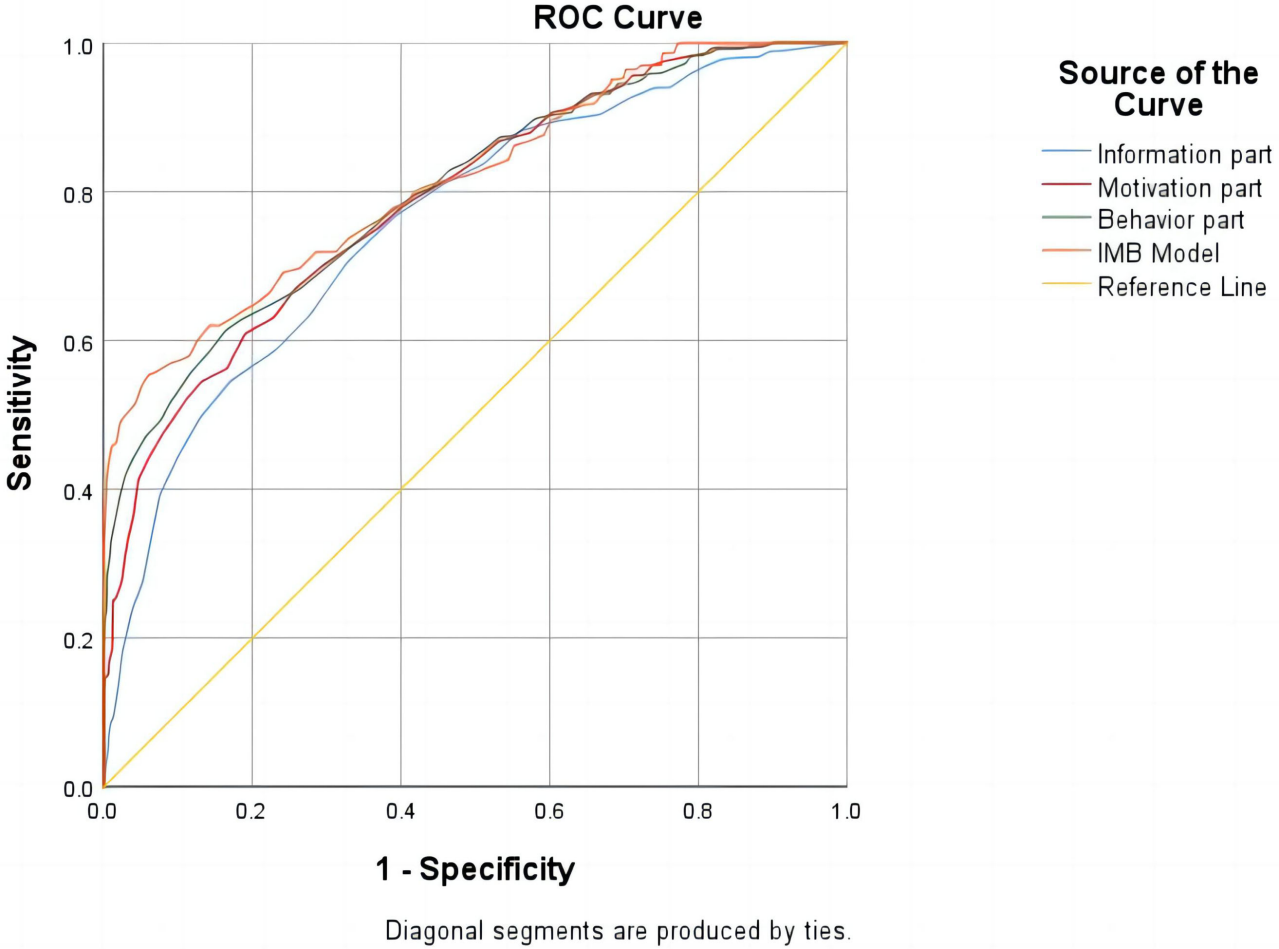
ROC curve of cumulative state on behavioral elements required for SMBG compliance with T2DM and pre-diabetes patients.

Receiver operating characteristics curve (ROC) analysis was aimed at evaluating prediction potential of IMB model. AUC represented the area under ROC curve, which indicated prediction accuracy. Its value was between 0 and 1, and the larger value was within the higher the accuracy. In the ROC curve, the best diagnostic cut-off point of SMBG compliance was when Youden index was the largest and the coincidence sensitivity was relatively high. In this study, SMBG compliance and non-compliance were taken as state variables of ROC, and the value of state variable was set as 1. The test variables were the total score of the scale and the scores of three sub-items (information, motivation and behavioral skills), respectively. The ROC curve analysis was carried out, and the relevant results were shown in **Figure 2**. The critical value of total IMB-SMBG score on adherence to SMBG was 136.5 points and AUC was 0.814.(95%CI=0.786～0.841, *P*<0.001); the critical value of subscale on information was 49.5 points, and AUC was 0.760 (95%CI=0.732~0.789, *P*<0.001); the critical value of subscale on motivation was 57.5points, and AUC was 0.790 (95% CI=0.762~0.817, *P*<0.001); the critical value of subscale on behaviors was 69.5points, and AUC was 0.800 (95% CI=0.773 ~ 0.828, *P*<0.001).

The results turned out that subscales on information, motivation and behavior skills all had high diagnostic value for SMBG compliance (AUC>0.7). For T2DM and pre-diabetes patients, the diagnostic accuracy of IMB-SMBG on behaviors was the highest, and the best diagnostic cut-off point was 69.5 points. Overall, the ROC curve showed superb prediction ability of IMB-SMBG (AUC = 0.814, 95%CI=0.786-0.841), which was beneficial to evaluate the potential of the final multivariate logistic regression model. When the total score of the three parts of the equivalence table is greater than or equal to 136.5 points, the blood glucose monitoring is up to standard. Specifically in the sub-items, it indicates that the intensity of patients’ daily information needs to be ≥49.5 points, the intensity of motivation needs to be ≥57.5 points, and the intensity of behavioral achievement needs to be ≥69.5 points. If a patient achieves these scores in SMBG, it indicates that the intensity requirements of information, motivation and practical behavior required by the patient to achieve the goal of daily blood glucose monitoring can promote the achievement of the goal of blood glucose monitoring. In addition, improving patients’ understanding and learning of diabetes knowledge, motivating them to take action, persuading them to believe and form good habits, and teaching them relevant behavioral skills can effectively promote the improvement of SMBG compliance to a certain extent.

## 4 Discussion

### 4.1 Status and problems of SMBG in T2DM patients

Results of this study showed that among 1388 patients with T2DM, only 26.2% (363/1388) patients reached SMBG standard. Given that SMBG is one of the individual predictors of type 2 risk in prediabetic patients, this result suggests low SMBG compliance. The results showed that patients with normal BMI had better SMBG compliance than those with abnormal BMI (OR=1.336, *P*<0.049). In T2DM patients, the rate of blood glucose monitoring in regular occupation was higher than that in non-regular occupation (OR=1.989, *P*<0.035). In terms of SMBG cognition, there is a significant difference between knowing SMBG frequency (*P*<0.001) and knowing SMBG’s sugar control goal (*P*<0.001). In terms of SMBG behavior, there are statistically significant differences in whether you feel pain at the acupuncture site (*P*<0.000), whether you can make up blood sugar test (*P*<0.005) and whether you can rotate your fingers during self-test (*P*<0.001). In terms of the feasibility of SMBG, there are statistically significant differences between whether it is convenient to buy a blood glucose meter/blood glucose test paper (P<0.047) and whether you know how to use all functions of the blood glucose meter (*P*<0.001).

### 4.2 Analysis of potential influencing factors and corresponding measures

#### 4.2.1 Accessibility and convenience of blood glucose meter/test paper

This study showed that T2DM patients who did not have convenient access to glucose meters or blood glucose test strips had worse SMBG compliance than those who did (*P*<0.047). The reason is that many T2DM patients cannot afford a blood glucose meter due to economic reasons (26). Although there are a variety of self-monitoring equipment and supplies for blood glucose in China, the price of blood glucose meter and blood glucose test paper is a certain pressure for patients with poor economic affordability, which leads to a low compliance of SMBG in some T2DM patients. From the perspective of objective factors, it is found that the lack of blood glucose meter is one of the obstacles of SMBG in diabetic patients (27). While patients with blood glucose meters tend to have higher frequency SMBG, because having blood testing equipment can makes it easy for patients to perform SMBG (28). Therefore, it would be remiss to ignore the economic impact and the accessibility and convenience of blood glucose meter/test paper, and the income of SMBG should be balanced with its cost. In this regard, we suggest that according to the characteristics of patients’ economic status and age, and refer to the suggestions of medical staff or patients’ families, help diabetic patients choose an economical blood glucose meter and teach them to use technology and some skills. For the manufacturers of glucose meters, we suggest that they develop new technologies to reduce the cost of blood glucose meters, ease the purchase pressure of diabetic patients, improve the accessibility of blood glucose meters, and encourage patients to perform regular SMBG.

#### 4.2.2 Cognition about SMBG

This study found that T2DM patients with good understanding of SMBG had better compliance with SMBG than those with poor understanding, that is, patients who knew the frequency of SMBG and clear glucose control goals had better compliance with SMBG, and the difference was statistically significant (*P*<0.001). The reason may be the misunderstanding of SMBG. Some patients think that blood glucose monitoring should be done by medical staff. They have monitored their blood glucose in hospital, but there is no need to monitor their blood glucose at home. Some patients think that SMBG is not helpful to control their own condition, so they don’t need to monitor their blood sugar when they feel good. Some patients are afraid that their blood glucose monitoring technology is not in place, and inaccurate blood glucose monitoring and incomplete disinfection will lead to infection and other misperceptions, resulting in poor compliance with SMBG (9). Studies have shown that T2DM patients have poor knowledge of SMBG (29), 54.09% of them do not know the frequency of SMBG, 95.08% of them do not know the correct time of SMBG, 67.62% of them do not know the goal of fasting blood glucose control, and 95.08% of them don’t know the goal of postprandial blood glucose control. Therefore, T2DM patients need to set a SMBG glucose control target, because goal setting has a certain positive effect on improving the short-term compliance of patients with SMBG (30), and it is also beneficial for patients to know about the positive aspects and potential negative aspects of SMBG, so as to further enhance the cognition of the positive aspects (31).

Postprandial blood glucose is also an important indicator of blood glucose control, but most T2DM patients only monitor fasting blood glucose, and do not know the significance of postprandial blood glucose, which is also the content of individualized diabetes education. Therefore, it is an important measure to develop individualized and targeted education and nursing program of diabetes blood glucose management to increase patients’ knowledge of blood glucose monitoring and compliance. In this regard, we suggest that medical staff should carry out more systematic, comprehensive and targeted SMBG guidance and education for patients according to the guidelines, so as to enhance their knowledge and belief of SMBG, improve their compliance of SMBG, and enable diabetic patients to actively and effectively conduct SMBG.

#### 4.2.3 Receive SMBG-related education

However, effective self-management requires frequent and high-level educational investment and continuous support. This study found that patients who actively know about medical health knowledge, such as T2DM patients who know about SMBG frequency and know how to use all functions of blood glucose meter (P<0.001), have better compliance with SMBG, which is consistent with previous research conclusions. In previous studies (32, 33), it was found that receiving blood glucose monitoring education can significantly improve SMBG compliance, and patients who have received diabetes education had better control of blood glucose level and glycosylated hemoglobin (34). T2DM patients who know how to use the blood glucose meter have better compliance with SMBG. The reason may be that patients who know how to use blood glucose meter pay more attention to their blood glucose level, so they will actively learn and understand some SMBG-related skills and knowledge to achieve the purpose of SMBG. The time point of SMBG and the goal of SMBG for T2DM patients vary from person to person, and are closely related to the age, course of disease, complications and drug use of patients. Diabetes education plays a very important role in the treatment of diabetes, which is related to the control of blood glucose and the prevention of complications. Therefore, in community diabetes patients, when the perfect disease management system is not well implemented, it is especially important for patients to actively learn and understand the knowledge of diabetes-related diseases to improve SMBG compliance.

Therefore, we suggest that T2DM patients establish a correct SMBG concept and receive SMBG-related education. First of all, the operation of SMBG is easy to learn, and it is not limited by time and place. Its measurement results can better reflect the blood glucose level of diabetic patients. For hospitalized patients, different forms of health education can be provided in the hospital, such as propaganda boards, brochures, small lectures, peer education (the missionary can effectively improve the effect of diabetes education and management by conducting the education as peers), etc. Secondly, for community patients, through community lecture halls, publicity materials, TV or pamphlets, the frequency, time and significance of SMBG and the correct operation method of blood glucose monitor are explained and demonstrated, so that T2DM patients can form a correct concept of SMBG. In addition, strengthening the education and management of patients’ SMBG and improving the level of patients’ SMBG are also inseparable from the education and management of medical staff. Medical staff should increase the number of SMBG drill teaching for patients, and focus on the misunderstandings that are easy to occur in SMBG during the drill (35).

#### 4.2.4 Relevant behaviors of SMBG

This study found that patients with T2DM who experienced needle pain had lower adherence to SMBG than patients who did not experience pain (P<0.001). The reason is that SMBG is a traumatic operation, and pricking the finger for sampling may induce certain negative effects. The pain related to finger pricking is probably the main reason why patients resist the use of SMBG, which makes them bored or even refuse to use SMBG. At the same time, there will be some consequences: such as scar formation and calluses; Loss of sensitivity, resulting in cognitive impairment (36). For acupuncture pain, we suggest to teach T2DM patients some techniques to relieve pain, such as telling patients to soak their hands in warm water before taking blood, choosing the angle of needle insertion according to the thickness of skin, choosing the right blood collection needle, and changing the blood collection needle every time. Through the application of new technology, the pain caused by needle pricking can be reduced. The use of SMBG gloves can reduce puncture infection, relieve the pain of diabetic patients during needle pricking, and improve the compliance of blood glucose monitoring. For example, the Scanning Glucose Monitoring FGM system, which consists of a flexible probe sensor, monitor, and related software, detects glucose concentrations in tissue with a flexible probe inserted under the skin. Finger blood calibration is not required during patient testing, which is minimally invasive, painless and easy to operate. Only scanning can obtain immediate glucose values and provide 14-day Ambulatory glucose Profile (AGP) (37). Through clinical application, it has been proved that it can effectively improve patients’ SMBG behavior, reduce the probability of hypoglycemia, and help patients reach the standard of SMBG frequency.

#### 4.2.5 Economic pressure

In a survey (38) of elderly diabetic patients, it was found that 89.5% of T2DM patients were reluctant to have their blood glucose monitored for economic reasons. At present, China’s blood glucose test paper has not been included in the scope of medical insurance reimbursement, which needs to be borne by patients themselves (39, 40). The direct cost of blood glucose monitoring is not high. The lowest price of a household blood glucose meter is less than that of 100 yuan, and the prices of blood collection needles and test paper are not high. The average monthly price is estimated to be as low as that of 30 yuan. However, due to daily consumption, it will still cause obvious economic burden to patients’ psychology. And because diabetes is a chronic lifelong disease, SMBG needs to be maintained for life. Blood glucose meter, blood glucose test paper and monitoring needle are expensive, and they are not covered by the reimbursement of urban medical insurance and rural cooperative medical insurance, which brings huge economic pressure to diabetic patients. Therefore, some families with poor economic status are reluctant to carry out SMBG in order to save medical expenses (41).

Therefore, on the one hand, medical staff need to strengthen patient health education, and inform patients that with the help of SMBG, medical staff can timely adjust the treatment plan according to the blood glucose control of T2DM patients, so that the blood glucose of patients can reach the standard, the occurrence of complications can be reduced, the additional medical expenses can be reduced, and patients can accept it voluntarily. On the other hand, we suggest that the medical insurance bureau can include test paper and needles in the scope of reimbursement, and limit a certain amount of reimbursement, thus alleviating the financial burden of diabetic patients.

Finally, the state or society should pay attention to diabetic patients and give psychological and spiritual support, so as to improve SMBG compliance of diabetic patients.

## 5 Conclusion

According to the analysis of this study, diabetes cognition, self-efficacy, glucose meter operation and monitoring cost of blood glucose meter are the potential influencing factors of SMBG in T2DM patients. Among them, understanding the frequency of SMBG and having a clear goal of sugar control were the promoting predictors of SMBG. In this study, the frequency of SMBG monitoring in T2DM patients still needs to be improved. Currently, there are still bottlenecks such as lack of disease health care knowledge and needle pain. In addition, the lack of accessibility and convenience of blood glucose meter and the heavy economic burden in T2DM patients are one of the barriers to the low frequency of SMBG monitoring, which further affects the effect of blood glucose control (42). In addition, forgetting to take a blood glucose test leads to a follow-up blood glucose test, and a 10-finger rotation during blood glucose test leads to higher SMBG compliance.

The low level of disease cognition can directly affect SMBG compliance of T2DM patients, and the lack of attention to blood glucose control makes it easy to ignore the exhortations of medical staff (43). Self-efficacy is reflected in patients’ confidence in adhering to blood glucose monitoring. Lack of self-efficacy makes it difficult for patients to believe that they can adhere to blood glucose monitoring for a long time, and it is easy to interrupt monitoring or reduce monitoring frequency (44). The operation of blood glucose meter is an important instrument for monitoring blood glucose level. Patients are unfamiliar with the operation of blood glucose meter, resulting in complex understanding of blood glucose monitoring and resistance. Social support is very important for patients’ SMBG. Some studies have shown that patients with better social support can better monitor their blood glucose under the supervision of others. Based on the above situation, hospitals should establish a long-term management mechanism for blood glucose monitoring, strengthen patients’ cognition of T2DM patients, and increase the importance of blood glucose monitoring. Strengthen individualized nursing intervention to improve patients’ self-efficacy; Guide patients to use blood glucose meters correctly and select appropriate blood glucose meters according to patients’ condition and economic conditions; Strengthen communication with patients’ families, and instruct them to actively supervise patients’ blood glucose monitoring.

## 6 Limitations and prospects

### 6.1 Limitations

This study is a cross-sectional study and could not find the relationship between SMBG and T2DM complications. A large-sample prospective cohort study should be conducted to explore the long-term benefits of SMBG on T2DM complications. There are many factors affecting SMBG, such as the comprehensive health status of patients and the degree of support from family members, which are not all included. Data collection was self-reported, so the data may be affected, such as memory bias. In addition, some patients may not truthfully answer the privacy questions in daily life, such as income. Due to some elderly people over 60 years old who didn’t use smart phones might not be surveyed in the questionnaire filled out by online platform, the majority of respondents were between 18 and 60 years old. This study did not include a glycemic index to assess glycemic control, such as fasting glucose values. And future research may focus on the relationship between SMBG compliance and glycemic control.

### 6.2 Novelty and prospect

Diabetes is a chronic lifelong disease that cannot be cured. It is impossible for patients to be treated in the hospital from the time of diagnosis of diabetes, and most of the time is self-management outside the hospital. Therefore, SMBG plays an important role in the blood glucose control of T2DM patients. With the development and popularization of blood glucose monitoring technology, SMBG has become the main form of blood glucose monitoring for diabetic patients. Standardized SMBG can make patients know whether their blood glucose is controlled at a good level, and provide effective basis for consulting doctors or timely adjustment of treatment plans.

The main advantage of this study lies in the cross-sectional design, which evaluates a series of variables, including gender, age, occupation, BMI, marital status, course of disease, pain, knowledge of SMBG frequency and glucose control goals, as well as basic demographic characteristics and living habits. This is helpful for us to explore the specific and highly detailed status of the quality of individual self-glucose monitoring in community T2DM population, and its relationship with individual information cognition, motivation and behavior.

The IMB model used in this study has practical value and extension in explaining SMBG of T2DM patients in Chinese communities. While behavior change is a continuous process, IMB model emphasizes that information, motivation and behavior skills are indispensable to complete behavior change. On the other hand, the research on SMBG compliance of diabetic patients based on IMB model can help patients improve their understanding of the disease, enhance their motivation for behavior change, and thus establish good self-management behavior habits of diabetes. This study makes up the gap between motivation and behavior shaping of T2DM patients’ health management behavior, provides new ideas for improving SMBG of diabetic patients, and provides direction and reference for improving self-management ability of diabetic patients, reducing or delaying the occurrence and development of complications. In addition, in future research, it would be beneficial to explore the differences in attention, application, and sophistication of SMBG devices among Type 2 diabetes populations in different regions of the world.

## Data availability statement

The raw data supporting the conclusions of this article will be made available by the authors, without undue reservation.

## Ethics statement

The studies involving human participants were reviewed and approved by Shantou University Medical College. Written informed consent to participate in this study was provided by the participants’ legal guardian/next of kin. The protocol was approved approved by The Ethics Committee of Shantou University Medical College (Code: SUMC-2021–064).

## Author contributions

ML undertook the literature reviews, data analysis, completion of statistical tables, research process, completion of result and discussion analysis. TC undertook data analysis, research process, completion of discussion and conclusion. GF was in charge of the conception design, undertook the design of the study framework and survey questionnaire, completed statistical tables and result analysis, took responsibility for the integrity of the data and the accuracy of the data and revised the entire manuscript. GF also wrote in the research process and interpreted the results, ultimately modified the manuscript. All authors read and approved the final manuscript.
